# Error-corrected next-generation sequencing mutagenicity assays in human HepaRG cells as human-relevant genetic toxicology new approach methodology

**DOI:** 10.3389/ftox.2025.1657189

**Published:** 2025-09-15

**Authors:** A. Rasim Barutcu, Nimisha Bhattarai, Raymond Samuel, Jamie Scaglione, Leslie Recio

**Affiliations:** ScitoVation, Durham, NC, United States

**Keywords:** NAM, mutagenecity, duplex-seq, ecNGS, HepaRG

## Abstract

Human metabolically competent HepaRG™ (HepaRG) cells have been developed as a human-relevant New Approach Methodology (NAM) in genetic toxicology, providing a non-animal alternative to rodent-based mutagenicity testing following a positive Ames test. Error-corrected next-generation sequencing (ecNGS) offers improved sensitivity, specificity, and mechanistic insight in genotoxicity and mutagenicity assessment.

**Methods:**

We applied duplex sequencing, an ecNGS approach, to quantify chemically induced point mutations in metabolically competent HepaRG cells. Cells were exposed to a diverse panel of genotoxic agents, including ethyl methanesulfonate (EMS), N-ethyl-N-nitrosourea (ENU), benzo[a]pyrene (BAP), cisplatin, cyclophosphamide, and etoposide. Mutation frequency, substitution patterns, and mutational signatures were analyzed, and results were compared with complementary cytogenetic endpoints.

**Results:**

Duplex sequencing detected dose-responsive increases in mutation frequency for ENU and EMS, with distinct substitution patterns consistent with alkylating mechanisms. BAP and cisplatin induced modest increases in mutation frequency and C>A-enriched spectra, while cyclophosphamide yielded minimal mutagenicity under the tested conditions. Etoposide triggered strong cytogenetic responses without increasing point mutations, consistent with its clastogenic mode of action. COSMIC mutational signature analysis revealed modest enrichment of SBS4 (BAP), SBS11 (EMS), and SBS31/32 (cisplatin), supporting the mechanistic relevance of the model.

**Discussion:**

These findings demonstrate the reproducibility and specificity of ecNGS for detecting low-frequency point mutations and characterizing mutational mechanisms. When combined with complementary cytogenetic assays, duplex sequencing enables a more complete and human-relevant evaluation of genotoxic potential. This study supports the integration of ecNGS into next-generation genotoxicity testing strategies as a NAM for regulatory decision-making.

## Introduction

The assessment of chemical mutagenicity is a cornerstone of regulatory toxicology, serving to protect public health by identifying agents capable of inducing heritable genetic changes that can lead to cancer, birth defects, and other adverse health outcomes. Historically, the regulatory framework for mutagenicity testing (ICH S2R1) has relied on bacterial reverse mutation assay, rodent cell chromosomal damage and gene mutation tests, and animal-based testing (National Research Council, 2007; Hartung, 2009). At present a human relevant mutagenicity assay is not required as part of the ICH S2 (R1) test battery. While these methods provide valuable information for risk assessment, they are considered to be hazard identification assays that are not useful for quantitative risk assessments and their relevance to humans has uncertainty (Rovida and Hartung, 2009; Chen et al., 2023).

There is a growing impetus to modernize toxicological testing paradigms by reducing reliance on animal models and adopting more predictive, human-relevant approaches (Chen et al., 2023). Legislative and regulatory changes, such as the 2016 amendment to the U.S. Toxic Substances Control Act (TSCA), now mandates the reduction and replacement of animal testing where scientifically reliable alternatives exist (Chen et al., 2023). Similarly, the European Union has banned the sale of animal-tested cosmetics, irrespective of the availability of alternative methods. On 10 April 2025, FDA published a roadmap outlining a “strategic, stepwise approach” for FDA to reduce, refine, and replace animal testing in preclinical safety studies with scientifically validated new approach methodologies (NAMs). However, the transition to non-animal NAMs requires robust qualifications to ensure that alternative assays accurately predict both traditional regulatory outcomes and, more importantly, human biological responses.

A critical unmet need in this context is the development of validated, human-relevant assays for the direct detection of mutagenicity. Genotoxicity assays, such as those measuring chromosomal aberrations or micronucleus formation are not measures of mutagenicity (OECD, 2016a; OECD, 2016b; OECD, 2023). Moreover, the mammalian cell mutation assay outlined in ICH S2(R1) relies on inbred rodent-derived, p53-deficient cell lines, which are prone to false-positive and lack human relevance (9). Human TK6 cells, which are p53-proficient and validated for use in genotoxicity assays (OCED, 2016a; OCED, 2016b), offer a promising alternative, but traditional mutation assays in these cells are laborious, low-throughput, and rarely employed in routine testing (Lutz, 1998; International Council for Harmonisation (ICH), 2011; Kirkland et al., 2005; Moore et al., 2002). Secondly, the lack of endogenous xenobiotic metabolizing enzymes limits the use of TK6 cells to assess risk to humans. Metabolically competent HepaRG cells provide a human-relevant, non-animal alternative to rodent-based mutagenicity assays and have been established as a genetic toxicology NAM for follow-up testing after a positive Ames result. Several publications measuring the same genetic toxicology endpoints employed in regulatory toxicology, the comet and micronucleus assays, the mode-of-action DNA damage transcriptome-based biomarker TGx DDI (Buick et al., 2020; Buick et al., 2022). More recently, we have focused on the induction of mutations using error-corrected next-generation sequencing (ecNGS) technologies. Our laboratory has been focused on HepaRG™ as a genetic toxicology multiple endpoint NAMs with seamless integration of multiple genetic toxicology endpoints from a single round of exposures that is aimed at reducing reliance on animals.

The advent of error-corrected next-generation sequencing (ecNGS), including duplex sequencing, represents a transformative advance in the field (Salk et al., 2018; Kinde et al., 2011; You et al., 2023). ecNGS enables the direct, high-sensitivity quantification of extremely rare mutational events (1 in 10^−7^ or lower) across the genome, bypassing the need for phenotypic expression time (7–8 days for hprt in TK6 cells), clonal selection, and dramatically reducing assay time (Chen et al., 2023; Salk et al., 2018; Stachler et al., 2023). Originally developed for detecting mutations *in vivo* (Schmitt et al., 2012; Izawa et al., 2023), ecNGS is now being adapted for mutagenicity assessment where it can quantify induced mutations from xenobiotic exposures and provide detailed mutational spectra and exposure-specific signatures (Valentine et al., 2020; LeBlanc et al., 2022). Recent studies have demonstrated strong concordance of ecNGS with gold-standard assays such as the transgenic rodent (TGR) assay and its ability to reveal mechanistic insights into chemical mutagenesis and clonal selection (Dodge et al., 2023; Ashford et al., 2025). Notably, several reports have extended ecNGS to human *in vitro* systems such as HepaRG™ and 3D liver spheroids (Seo et al., 2023), illustrating its translational potential and relevance to New Approach Methodologies. Despite this promise, the successful integration of ecNGS into regulatory toxicology requires continued development of standardized protocols, demonstration of reproducibility across labs, and validation within defined human-relevant test systems, and integration of ecNGS into the OECD test guidance (Marchetti et al., 2023).

The current project addresses this need by developing and validating ecNGS-based mutagenicity assays in metabolically competent HepaRG™ cells, aiming to provide a medium-throughput, human-relevant alternative to traditional *in vitro* and *in vivo* assays. This approach is poised to fill a critical data gap in the genetic toxicology test battery human relevant gene mutation assay, reduce animal use, and enable more accurate, efficient, and mechanistically informative safety assessments for pharmaceuticals, industrial chemicals, and environmental contaminants (Kucab et al., 2023).

## Materials and methods

### Chemicals

Test chemicals were purchased from Sigma-Aldrich (St. Louis, Missouri, United States) for exposures in human cryopreserved No-Spin HepaRG™ cells (Triangle Research Labs (TRL), Durham, North Carolina, United States). The chemical exposures in HepaRG™ cultures and the paired high-content flow cytometry data were conducted at ScitoVation (6 Davis Dr, Durham, North Carolina, United States).

### HepaRG™ cell culture and chemical exposures

Differentiated No-Spin HepaRG™ cells were cultured and exposed in a 2D format (Lonza, Durham NC). Briefly, No-Spin HepaRG™ cells were seeded at approximately 4.8 × 10^5^ viable cells per well in 24-well collagen coated plates in Lonza Thawing and Plating Medium for 24 h, then switched to Lonza Pre-Induction/Tox supplemented medium for cell maintenance and treatment (Lonza, Durham NC). The 24-well plate format was chosen in order to provide sufficient numbers of cells per replicate for the cytotoxicity and MN assay and other downstream endpoints (e.g., transcriptomics) without the need to pool wells. Cells were incubated for 7 days following seeding to allow the cells to regain peak metabolic function (Aninat et al., 2006; Lonza, Durham NC). A range finder in a 96 well format was done prior to conducting of the MN assay in 24-well plates, each concentration was tested in triplicate. Cells were exposed for 24 h then exposure test articles were removed, media was re-freshed and the cells were stimulated with human Epidermal Growth Factor-1 (hEGF) for an additional 72 h to induce cell division. Cell were then transferred (sub-cultured) to 12-plates for 48 h in Lonza Pre-Induction/Tox supplemented medium then stimulated with hEGF to induce a second round of population doubling over 48 h. These two rounds of hEGF mitogen stimulation were found to increase the cell population by approximately 2-fold each time. The methods outlined in Buick et al. (2020), were used to assess the frequency of cytotoxicity and micronuclei in HepaRG™ vehicle controls and from HepaRG™ test compound exposures. At the end of second hEGF stimulation the cells were harvested for DNA isolations. Three genotoxic compounds that require metabolic biotransformation by at least two distinct CYP450s to produce genotoxic compounds were tested. Benzo(a)pyrene that is bioactivated to genotoxic metabolites by CYP1A1, cyclophosphamide bioactivated to genotoxic metabolites by CYP3A4, and the known human liver carcinogen aflatoxin B1 bioactivated by CYP3A4. Each of these positive controls induced a dose-dependent increase in cytotoxicity and the induction of MN in HepaRG cells.

### *In Vitro* MicroFlow cytotoxicity assay

The flow cytometry-based cytotoxicity and MN assay was performed using the *In Vitro* MicroFlow kit (Litron Laboratories, Rochester, New York, United States) as described in Buick et al. (2020). Data and details reported in Recio et al. (2025, *manuscript in preparation*). Data were collected using a Miltenyi Biotec MACSQuant Analyzer 10 with an automated 96 well sampler (Auburn, CA). Unless precluded by test article precipitation or excessive cytotoxicity ∼5,000 cells were analyzed to determine relative survival (% RS) and the MN frequency (% MN). A detailed description of the methods is outlined (Buick et al., 2020). In brief, % RS was determined using nuclei-to-bead ratios in exposed versus control cells by spiking in counting beads to the cell suspensions to function as the internal standards. Cytotoxicity were not analyzed statistically but was used to set a “cut-off” of approximately 50% survival to identify exposure concentrations to process for DNA extractions and DupSeq library prep. Cytotoxicity data are expressed as the mean ± standard deviation (SD) from one definitive experiment with concentrations tested in triplicate; exposure concentrations used in the definitive experiment were based on a previously tested in a range finder experiment.

### DNA extraction and quality assessment

Genomic DNA was extracted from tissue samples using the DNeasy Blood & Tissue Kit (Qiagen, Hilden, Germany) according to the manufacturer’s protocol. DNA concentration was measured using a Qubit 4.0 fluorometer with the dsDNA HS Assay Kit (Thermo Fisher Scientific, Waltham, MA, United States). DNA integrity was assessed by agarose gel electrophoresis, and only samples with high molecular weight DNA showing minimal degradation were used for downstream analysis.

### TwinStrand duplex sequencing library preparation

TwinStrand duplex sequencing libraries were prepared using the TwinStrand Mutagenesis Kit (TwinStrand Biosciences, Seattle, WA, United States) following the manufacturer’s recommended protocol. Briefly, 500 ng of high-quality genomic DNA was ultrasonically sheared to a mean fragment size of approximately 300 bp using a Covaris M220 focused ultrasonicator (Covaris, Woburn, MA, United States). Fragmented DNA was end-polished and A-tailed using the manufacturer’s protocols.

TwinStrand duplex adapters containing unique molecular identifiers (UMIs) were ligated to DNA fragments using T4 DNA ligase (New England Biolabs). The adapter-ligated DNA was subjected to size selection using AMPure XP beads (Beckman Coulter, Brea, CA, United States) to remove adapter dimers and select fragments in the 250–400 bp range. Library amplification was performed using Q5 High-Fidelity DNA Polymerase (New England Biolabs) with TwinStrand-specific primers for 12–15 PCR cycles to minimize amplification bias.

### Target enrichment and quality control

Libraries were subjected to hybrid capture enrichment using custom-designed biotinylated RNA baits targeting specific genomic regions of interest. Enrichment was performed using the SeqCap EZ Prime Choice Library (Roche, Basel, Switzerland) according to the manufacturer’s protocol. Post-enrichment libraries were quantified using qPCR with the KAPA Library Quantification Kit (Roche) and fragment size distribution was analyzed using the Agilent 2100 Bioanalyzer with the High Sensitivity DNA Kit (Agilent Technologies, Santa Clara, CA, United States).

### Sequencing and data generation

Paired-end sequencing (2 × 150 bp) was performed on an Illumina NovaSeq 6000 platform (Illumina, San Diego, CA, United States) using the NovaSeq 6000 S4 Reagent Kit. Libraries were pooled to achieve a target depth of >1,000× unique duplex coverage per sample. Raw sequencing data were demultiplexed using bcl2fastq2 (Illumina) and quality metrics were assessed using FastQC v0.11.9.

### Duplex sequencing and analysis

DNA libraries underwent hybrid capture using custom biotinylated RNA baits (Roche) and were sequenced on an Illumina NovaSeq 6000 to a depth of >1,000× duplex coverage. Sequencing reads were processed using the TwinStrand DuplexSeq™ Mutagenesis App (v3.11.0) on the DNAnexus platform (LeBlanc et al., 2022). The pipeline includes UMI-based read collapsing, duplex consensus sequence (DCS) generation, alignment to GRCh38, variant calling, and filtering using TwinStrand’s high-fidelity mutagenesis pipeline. Only variants supported by DCS coverage ≥100× and meeting predefined strand bias and background error thresholds were retained. Mutation frequencies and spectra were calculated across target regions. Mutational signature extraction and attribution were performed using COSMIC v3.4 reference signatures. Signature contributions were validated by cosine similarity and bootstrapped confidence intervals. Statistical analysis: Mutation frequencies are shown as means ±95% confidence intervals based on biological replicates; no p-values were calculated due to the low event rates and focus on trend consistency.

## Results

### Mutation frequency stability across historical controls

Basal mutation frequencies in human cell systems can fluctuate over time due to intrinsic replication dynamics, clonal selection, or environmental drift. To evaluate the long-term reproducibility of the EC-DNS mutagenesis assay and establish a benchmark for chemically induced signal detection, untreated HepaRG™ cells were processed across independent experimental rounds, spanning multiple weeks and reagent lots.

**FIGURE 1:**
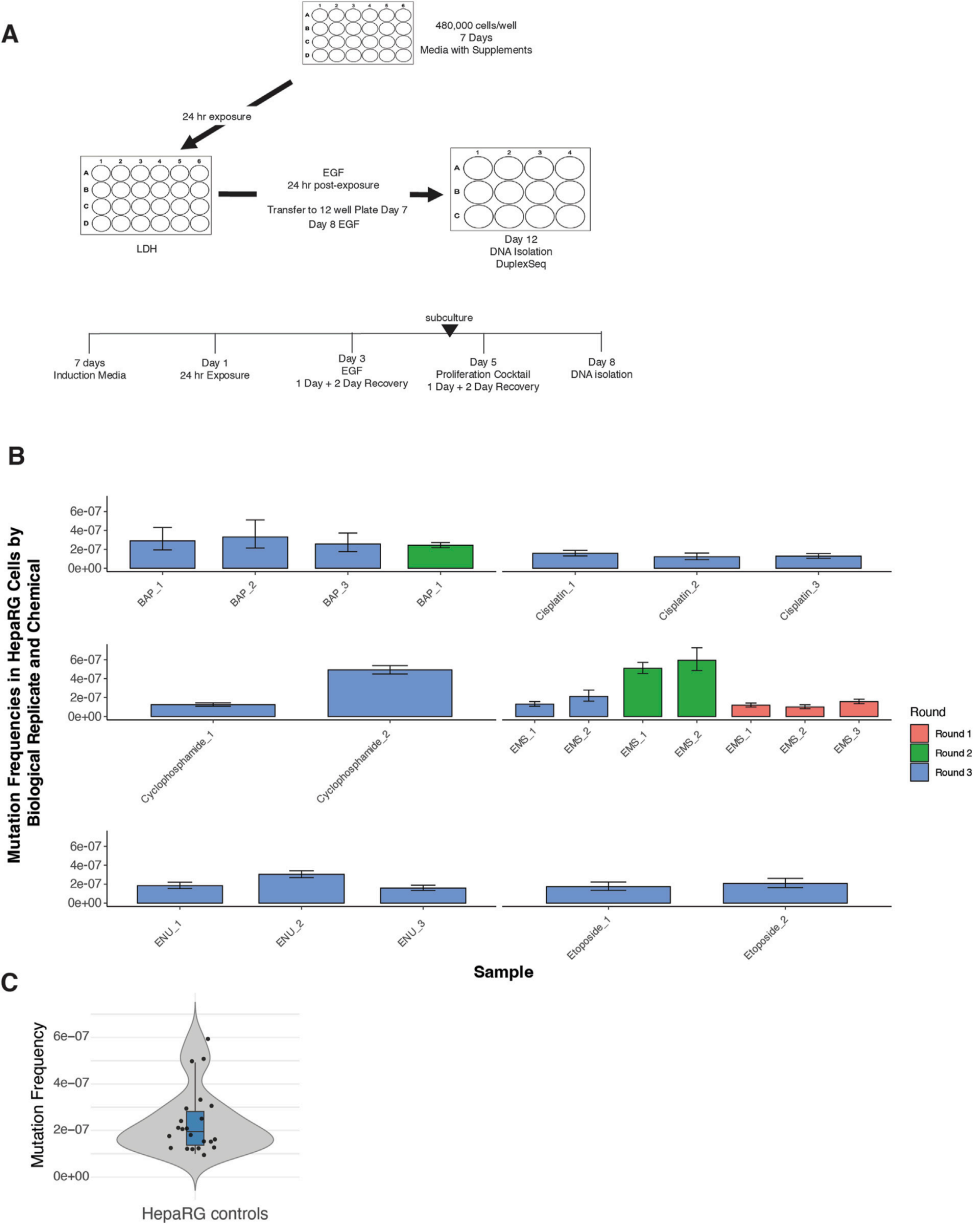
Experimental design and background mutation frequencies in HepaRG™ cells using ecNGS. **(A)** Schematic of the ecNGS exposure and analysis protocol. Differentiated HepaRG™ cells were treated with test chemicals for 24 h in 2D culture, followed by mitogenic stimulation to fix mutations, with DNA isolated on Day 12 for duplex sequencing. **(B)** Mutation frequencies from single-dose chemical exposures compared to vehicle-treated controls, aggregated across three experimental rounds. The number of biological replicates varies by compound due to phased study design: EMS was tested across three rounds, BAP across two, and others (e.g., etoposide, cisplatin) in a single round. Bars show mean ±95% confidence intervals for each replicate. **(C)** Violin plot summarizing mutation frequency distribution in all DMSO vehicle control samples across the study timeline. The violin shape shows full distribution; box limits indicate the interquartile range (IQR), the horizontal line denotes the median, and whiskers extend to 1.5× IQR.

The experimental design incorporated synchronized plating, defined exposure windows, and sequential mitogenic stimulation to drive replicative fixation of DNA lesions (Figure 1A). A total of 20 vehicle control samples were processed across the full study timeline.

Mutation frequencies from single-dose chemical exposures were benchmarked against the historical control distribution (Figure 1B). For the majority of tested compounds—including ENU, EMS, cisplatin, benzo[a]pyrene, and etoposide—replicate-level mutation frequencies closely aligned with background values, showing little evidence of technical or biological drift across rounds. An exception was noted for cyclophosphamide in Round 3, where one replicate displayed an elevated mutation frequency and a broader confidence interval. However, the other replicates for this compound remained consistent with control levels, suggesting an isolated variation rather than systematic assay deviation. Furthermore, background mutation frequencies in untreated HepaRG™ cells were low and tightly distributed across replicates (median MF ≈ 2.5 × 10^−7^) (Figure 1C), supporting high reproducibility of the assay pipeline.

Together, these results confirm the stability of background mutagenesis in the HepaRG™ system and support the validity of this model for comparative mutagenicity screening using EC-NGS.

### Ethyl methanesulfonate (EMS) and N-ethyl-N-nitrosourea (ENU) exposure

To evaluate the ability of DuplexSeq to quantify dose-responsive mutagenesis, HepaRG cells were treated with ethyl methanesulfonate (EMS) or N-ethyl-N-nitrosourea (ENU) across a range of concentrations followed by EC-NGS analysis of mutation frequency and signature profiles.

**FIGURE 2:**
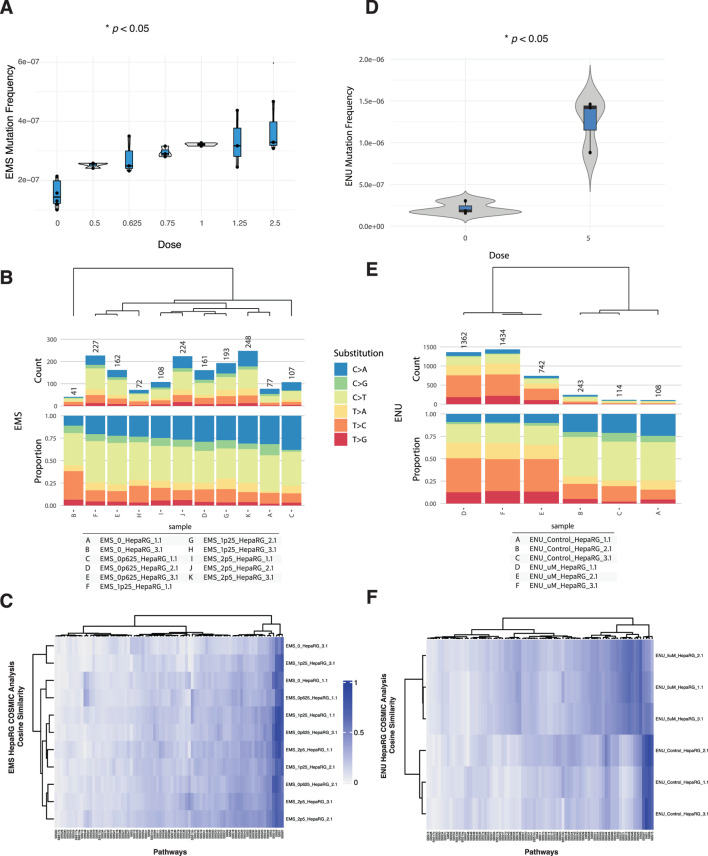
Mutagenicity of EMS and ENU in HepaRG™ cells assessed by ecNGS and COSMIC signature profiling. **(A)** Mutation frequencies in cells exposed to increasing concentrations of EMS. Error bars indicate 95% confidence intervals. **(B)** Base substitution spectra for EMS-exposed cells shown as proportional stacked barplots by substitution class (C>A, C>G, C>T, T>A, T>C, T>G). **(C)** COSMIC mutational signature heatmap for EMS-treated samples showing modest enrichment in signatures associated with alkylation and base excision repair deficiency. **(D)** Mutation frequency in HepaRG™ cells following exposure to 5 μM ENU, demonstrating elevated mutation levels relative to controls. **(E)** Base substitution spectra in ENU-exposed and vehicle-treated samples showing increased C>T transitions. **(F)** COSMIC signature similarity heatmap for ENU-exposed cells showing modest enrichment of alkylating agent–related signatures.

Mutation frequency analysis revealed no appreciable increase in mutagenesis across the EMS dose series up to 2.5 mM (Figure 2A). Individual replicates at each dose clustered tightly and overlapped the background distribution established in control cells. This finding was consistent across three independent rounds of EMS exposure and analysis. Likewise, base substitution spectra across the EMS dose series were similar to controls, with the dominant substitution class remaining C>T transitions at similar proportions across all conditions (Figure 2B).

In contrast, ENU exposure at 5 µM produced a clear elevation in mutation frequency relative to matched vehicle controls (Figure 2C). The increase was reproducible across three replicates and accompanied by a shift in mutational spectra. ENU-exposed samples showed elevated C>T transitions, along with a broader distribution of substitution types compared to vehicle-treated cells (Figure 2D). These observations are consistent with the known DNA alkylating properties of ENU (Hoyos-Manchado et al., 2020).

Micronucleus formation and relative survival were also quantified for both agents (Supplementary Figures S1A, S2A). EMS exposures above 1.25 mM elicited modest increases in micronuclei alongside decreased relative survival, while ENU exposures across the tested range produced minimal cytotoxicity and micronucleus induction.

To explore the mutational processes induced by these agents, we compared the trinucleotide context spectra of exposed samples to established COSMIC single-base substitution (SBS) signatures using cosine similarity analysis (Alexandrov et al., 2020). The results, shown in Supplementary Figures S1B, S2B, indicate that most EMS- and ENU-treated samples retained high similarity to background profiles, with subtle shifts in specific signature weights.

For EMS, modest increases in similarity were observed to SBS1 (associated with endogenous deamination), SBS30 (base excision repair deficiency), and SBS7b (UV-related damage), though none of these dominated the spectra. These may reflect subtle shifts in cellular processing of EMS-induced lesions or background processes exacerbated under stress. No definitive attribution to a single repair-deficient or exogenous damage pathway was apparent.

ENU-treated samples showed limited but measurable alignment with SBS32 (azathioprine exposure), SBS31 (platinum treatment), and SBS1. The consistency of these similarities across replicates was moderate, and none reached levels suggestive of unambiguous pathway attribution. As with EMS, these enrichments may reflect convergent substitution profiles from alkylating stress.

Taken together, these results confirm the capacity of DuplexSeq to detect dose-dependent mutagenesis for potent alkylating agents like ENU, while also illustrating the importance of contextual interpretation for COSMIC deconvolution, especially when mutagenic signals remain close to baseline.

### Benzo[a]pyrene (BAP) and cyclophosphamide (CPA) exposure

To evaluate the genotoxicity of benzo[a]pyrene (BAP) and cyclophosphamide (CPA) in HepaRG cells, we assessed cytotoxicity, micronucleus (MN) formation, mutation frequency, and COSMIC mutational signature profiles across biologically independent replicates.

In the MN assay, BAP exposure induced a modest, dose-dependent increase in micronucleus frequency beginning at 25 μM, accompanied by a small decrease in relative survival at the highest doses tested (Supplementary Figure S3A). In contrast, CPA exposure led to a sharper increase in MN formation at 5 and 10 mM, with a decrease in survival by ∼50% at the highest dose (Supplementary Figure S4A).

**FIGURE 3:**
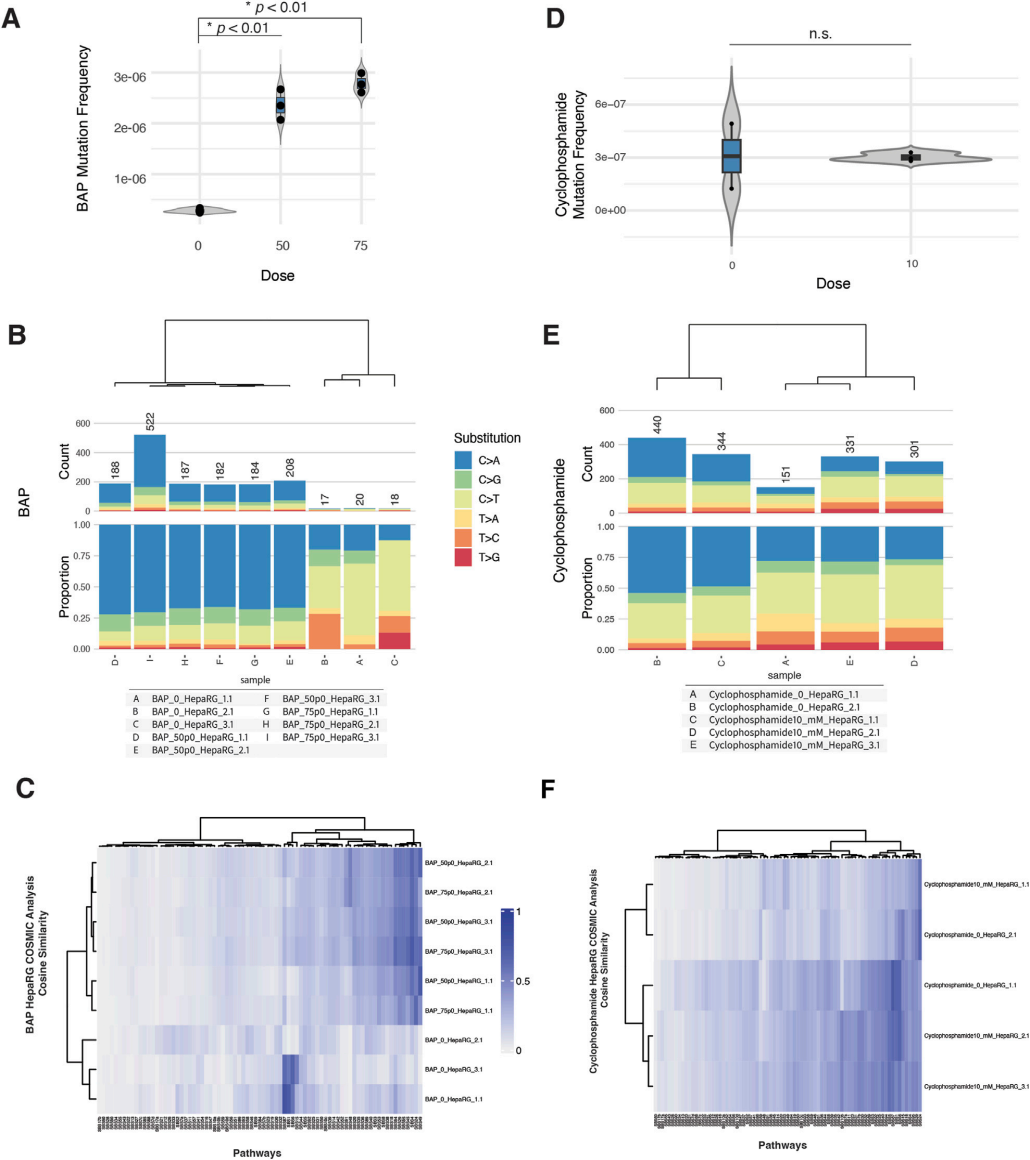
ecNGS and COSMIC profiling of mutagenic responses to benzo[a]pyrene (BAP) and cyclophosphamide (CPA). **(A)** Mutation frequencies in HepaRG™ cells after exposure to 50 and 75 μM BAP. **(B)** Base substitution spectra for BAP-treated samples showing predominance of C>A mutations, consistent with bulky adduct formation. **(C)** COSMIC signature enrichment heatmap for BAP-treated replicates, showing similarity to SBS4 and SBS29 (PAH-related mutational processes). **(D)** Mutation frequencies in cells exposed to 10 mM CPA, showing no consistent elevation over control levels. **(E)** Substitution spectra for CPA-exposed samples compared to background. **(F)** COSMIC signature similarity in CPA-treated samples, with weak enrichment in SBS5 and SBS87 (clock-like and thiopurine-associated processes).

Despite these cytogenetic effects, mutation frequency remained low across most conditions. For BAP, exposures at 50 and 75 µM produced consistent increases in mutation frequency relative to controls (Figure 3A). Base substitution spectra across BAP-treated samples were dominated by C>A transversions (Figure 3B), consistent with BAP’s established mechanism of inducing bulky DNA adducts and subsequent transversion-type mutations (Khalili et al., 2000).

In contrast, CPA treatment at 10 mM did not result in a significant elevation in mutation frequency compared to untreated controls (Figure 3C). Mutation spectra for CPA-exposed cells were similar to background, with no evident shift in substitution profile (Figure 3D).

COSMIC signature analysis provided additional context. BAP-exposed samples showed modest but reproducible enrichment of SBS4 (associated with tobacco smoking) and SBS29 (linked to tobacco chewing), both characterized by C>A-rich mutation patterns (Supplementary Figure S3B). These findings are consistent with known BAP-associated mutational processes but do not reflect dominant signature activity (Hoyos-Manchado et al., 2020). CPA-treated samples exhibited minor similarity increases to SBS5 (clock-like, etiology unknown) and SBS87 (thiopurine chemotherapy–associated), although these did not reach strong cosine similarity values and varied between replicates (Supplementary Figure S4B).

Taken together, these data suggest that BAP can induce weak but detectable mutagenic effects in HepaRG cells, accompanied by modest C>A-biased mutation spectra and signature enrichments. CPA, despite evidence of clastogenicity in MN assays, produced little evidence of sequence-level mutagenesis under these assay conditions.

### Etoposide and cisplatin exposure

In micronucleus assays, etoposide exposure produced a dose-dependent increase in % micronuclei beginning at 6.25 µM, with a sharp rise at 12.5 and 25 μM, coinciding with reduced relative survival (Supplementary Figure S5A). Cisplatin exposure also increased % micronuclei beginning at 6.25 µM, with a concurrent drop in survival, indicating cytogenotoxic stress (Supplementary Figure S6A).

**FIGURE 4:**
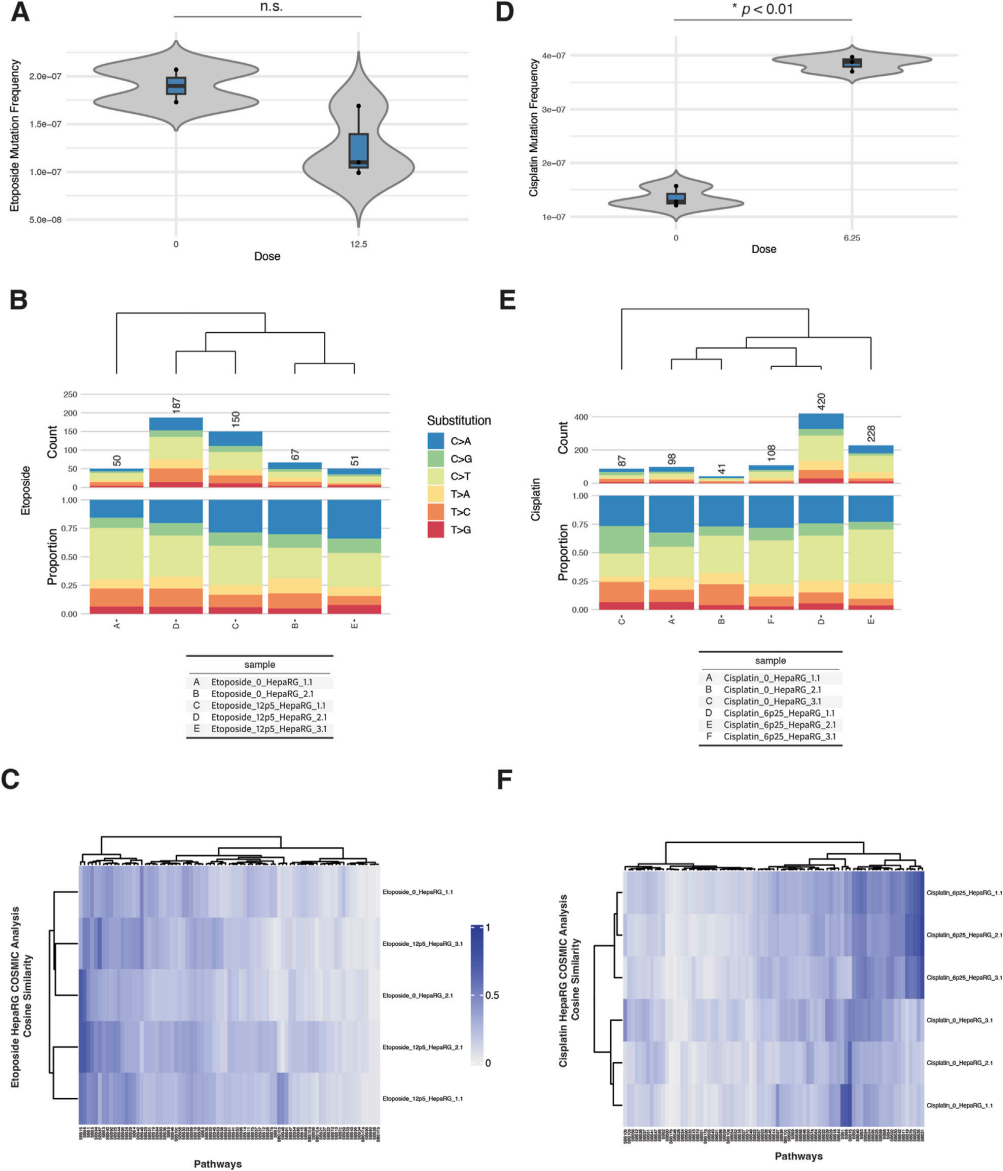
ecNGS analysis of etoposide and cisplatin mutagenicity and COSMIC signature associations in HepaRG™ cells. **(A)** Mutation frequencies in HepaRG™ cells exposed to 12.5 μM etoposide. Bars show mean ±95% confidence intervals for each replicate. **(B)** Base substitution spectra for etoposide-treated and control samples, dominated by C>T transitions. **(C)** COSMIC signature heatmap showing minor enrichment for mismatch repair–associated signatures (e.g., SBS15, SBS6) in etoposide-exposed cells. **(D)** Mutation frequencies following 6.25 μM cisplatin exposure, with a slight elevation over controls. **(E)** Base substitution patterns in cisplatin-treated cells, showing no dominant mutation class. **(F)** COSMIC signature similarity heatmap showing low-level enrichment for platinum-associated signatures SBS31 and SBS32 in cisplatin-exposed cells.

Despite these cytogenetic effects, EC-NGS analysis revealed only modest increases in mutation frequency. Etoposide-treated cells at 12.5 µM displayed mutation frequencies that overlapped the control range across replicates (Figure 4A). Substitution spectra showed C>T transitions as the dominant class across all etoposide-treated and control samples, without a pronounced shift in spectrum profile (Figure 4B).

Cisplatin exposure at 6.25 µM resulted in a slight but consistent elevation in mutation frequency above control (Figure 4C). Base substitution profiles remained broadly similar between exposed and unexposed conditions, with no emergence of a dominant mutational class (Figure 4D).

COSMIC signature analysis revealed weak but reproducible increases in similarity to certain reference signatures. Etoposide-exposed samples showed low-level enrichment for SBS15 (defective mismatch repair), SBS6 (MMR-deficient signature), and SBS87 (associated with thiopurine treatment), though these similarities were modest and did not dominate any sample (Supplementary Figure S5B). Cisplatin-treated cells showed minor increases in similarity to SBS31 (platinum chemotherapy), SBS32 (azathioprine exposure), and SBS87, with variability across replicates (Supplementary Figure S6B). These signatures are biochemically plausible given the DNA adduct-forming and cross-linking nature of cisplatin, but observed cosine similarities were low-to-moderate.

Taken together, etoposide and cisplatin produced detectable cytogenetic effects and weak increases in mutation frequency in HepaRG cells. COSMIC analyses identified plausible but non-dominant signature enrichment patterns consistent with known or chemically related mechanisms.

## Discussion

In this study, error-corrected next-generation sequencing (ecNGS) using duplex sequencing demonstrated high sensitivity and specificity for quantifying chemically induced point mutations in metabolically competent HepaRG cells. Chemicals representing a range of genotoxic mechanisms—including the alkylating agents ENU and EMS, the polycyclic aromatic hydrocarbon benzo[a]pyrene (BAP), and the DNA crosslinker cisplatin—produced clear, reproducible increases in mutation frequency, often accompanied by distinct base substitution spectra. In contrast, the pro-mutagen cyclophosphamide yielded a minimal mutagenic response, and the topoisomerase II inhibitor etoposide elicited strong cytogenetic effects without measurable point mutagenesis. These compound-specific profiles illustrate the mechanistic diversity captured by ecNGS and support its integration as a human-relevant tool within next-generation genotoxicity assessment frameworks.

Mutation spectra derived from duplex sequencing reflected mechanistic specificity. ENU and EMS exposures generated C>T-rich profiles consistent with alkylation-induced transitions, aligning with COSMIC mutational signatures associated with alkylator treatment (e.g., SBS11). BAP exposure was associated with increased C>A transversions, a hallmark of bulky DNA adduct formation, and modest enrichment of SBS4 and SBS29, signatures observed in tobacco-related cancers. Cisplatin induced modest shifts in mutational spectra, with low-level similarity to platinum-related signatures (e.g., SBS31, SBS32), though the mutation frequency increase was limited. Notably, cyclophosphamide produced minimal point mutagenesis under the assay conditions, likely reflecting incomplete metabolic activation in this *in vitro* system or repair of generated lesions before fixation. Etoposide, while clastogenic as evidenced by increased micronucleus frequency, did not induce measurable point mutations, consistent with its known mode of action involving double-strand breaks and large deletions rather than single-base substitutions.

These findings underscore ecNGS’s ability to directly detect point mutations and identify compound-specific mutational fingerprints. Beyond mutation frequency, the capacity to resolve mutational spectra provides a mechanistic layer of interpretation, increasing the assay’s relevance for hazard identification and mode-of-action analysis. This is consistent with recent applications of ecNGS in both *in vitro* and *in vivo* systems, which have demonstrated its ability to recover exposure-specific mutational signatures with high fidelity, including for BAP and other carcinogens (Valentine et al., 2020; LeBlanc et al., 2022; You et al., 2023).

Moreover, the high reproducibility and low background noise of duplex sequencing permit the detection of low-frequency mutations with statistical confidence—an important attribute for distinguishing weak mutagens from non-mutagens in regulatory contexts. Comparative studies have shown that ecNGS-based methods, including DuplexSeq, outperform traditional transgenic rodent assays in both sensitivity and precision, while requiring fewer animals and replicates due to lower inter-sample variability (Dodge et al., 2023; Ashford et al., 2025).

The performance of ecNGS also highlights its complementarity with traditional genotoxicity assays. The divergent profiles of ENU and etoposide exemplify how combining endpoints yields a more complete picture of genotoxic liability. ENU, a potent point mutagen, triggered minimal micronucleus formation but strong mutation frequency increases in ecNGS, whereas etoposide triggered robust clastogenicity without base substitution signatures. This supports a tiered or integrated testing strategy in which assays such as ecNGS, micronucleus, comet, and γH2AX are applied in parallel to detect a broad spectrum of DNA damage modalities.

From a NAM and regulatory perspective, this study contributes to the growing body of evidence supporting the utility of ecNGS as a quantitative, human-relevant alternative to legacy animal-based mutagenicity tests. HepaRG cells offer an advantageous test system due to their metabolic competence and stable karyotype, allowing both direct- and pro-mutagens to be evaluated. The observed concordance between *in vitro* mutation signatures and known COSMIC signatures from human cancers further reinforces the translational value of this system. Such mechanistic anchoring to human disease pathways is increasingly important for next-generation risk assessment, particularly in weight-of-evidence frameworks or adverse outcome pathway (AOP) contexts (Salk et al., 2018; Marchetti et al., 2023).

Despite its advantages, ecNGS has practical limitations that must be addressed before regulatory adoption. First, its focus on point mutations excludes larger structural variants, copy number alterations, and chromosomal aberrations—important damage types captured by cytogenetic assays. Second, while costs are decreasing, ecNGS remains more resource-intensive than simpler assays, posing a barrier to high-throughput screening or broad regulatory deployment. Third, harmonized protocols and reference datasets are still needed to support inter-laboratory reproducibility and facilitate interpretation across diverse chemical classes and cell systems. Ongoing efforts by regulatory working groups and consortia (e.g., HESI, OECD, NC3Rs) are beginning to address these gaps through collaborative validation studies and protocol standardization initiatives. Future studies will expand the chemical test set to include additional agents that induce chromosomal damage and require complex metabolic activation, enabling a more comprehensive evaluation of assay performance across diverse genotoxic mechanisms.

Overall, the data presented here support the role of duplex sequencing as a powerful addition to the genotoxicity testing toolbox. When paired with complementary endpoints, ecNGS enables a more nuanced and mechanism-informed evaluation of genotoxic potential—one that is better aligned with human biology, sensitive to low-level events, and adaptable to diverse testing scenarios. Its implementation within regulatory frameworks, particularly as a NAM for *in vitro* mutagenicity assessment, will depend on continued method development, cross-laboratory validation, and demonstration of fit-for-purpose utility. As the field moves toward modernized safety assessment strategies that emphasize relevance, reliability, and reduction of animal use, ecNGS stands out as a scientifically robust approach that can enhance both mechanistic understanding and regulatory confidence in mutagenicity decision-making.

## Data Availability

The raw sequencing reads generated in this study have been deposited in the NCBI Sequence Read Archive (SRA) under BioProject accession number PRJNA1306197.
