# Supplementary figures and images for: Error-corrected next-generation sequencing mutagenicity assays in human HepaRG cells as human-relevant genetic toxicology new approach methodology
Source: Front Toxicol. 2025 Sep 15;7:1657189. doi: 10.3389/ftox.2025.1657189 (PMC12477553; doi:10.3389/ftox.2025.1657189)

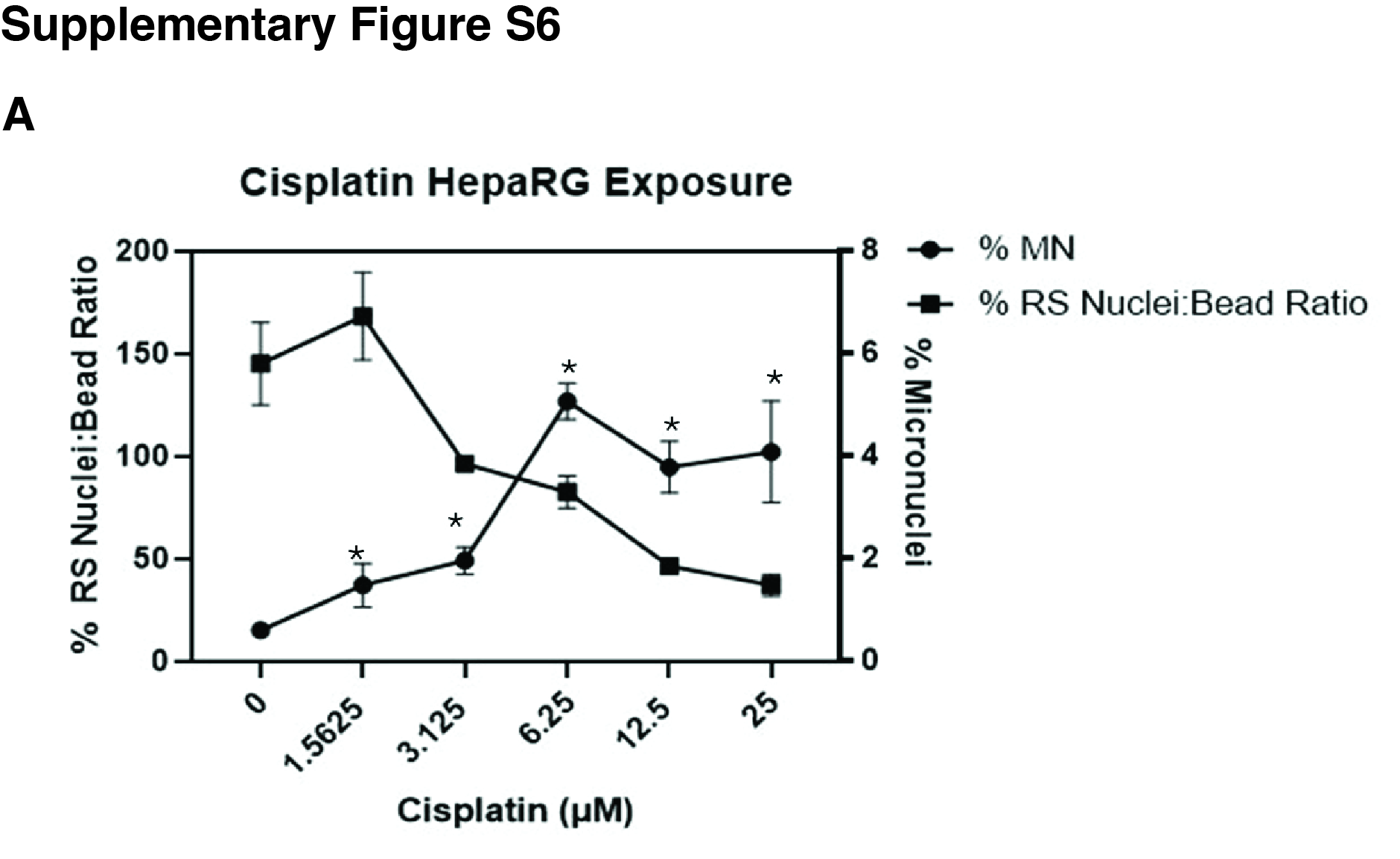

Supplement: Supplementary file 1 [file Image6.tif]

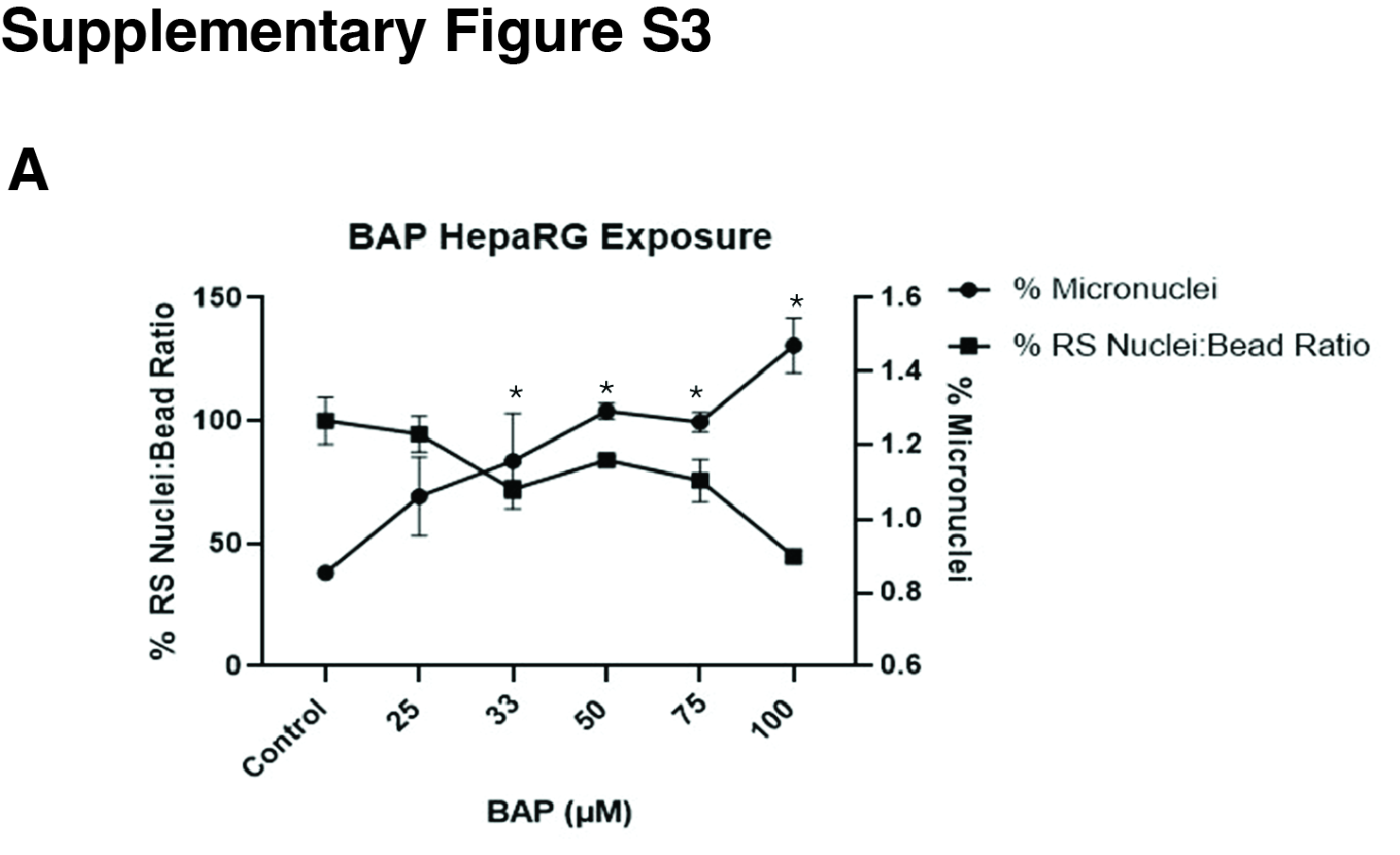

Supplement: Supplementary file 2 [file Image3.tif]

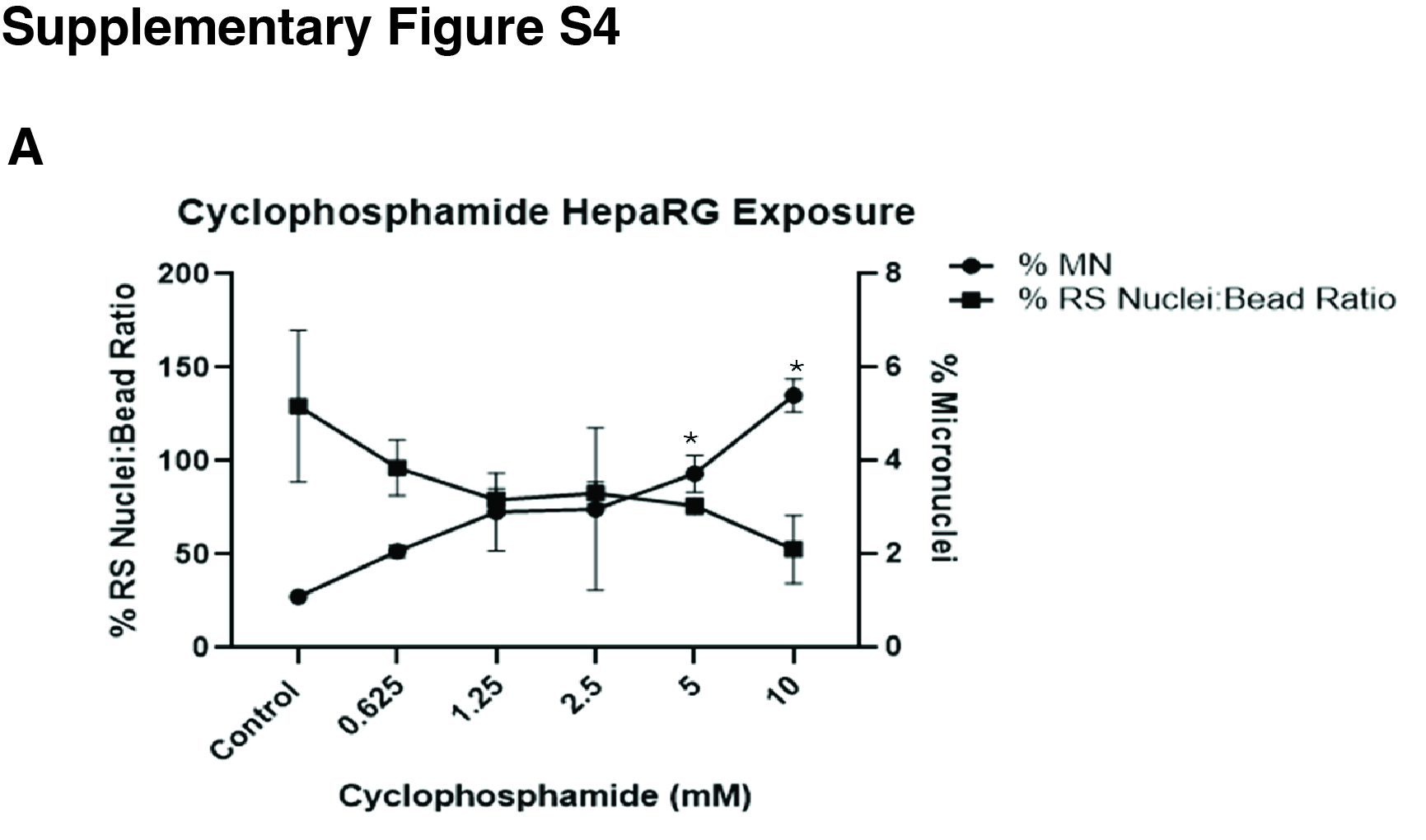

Supplement: Supplementary file 3 [file Image4.tif]

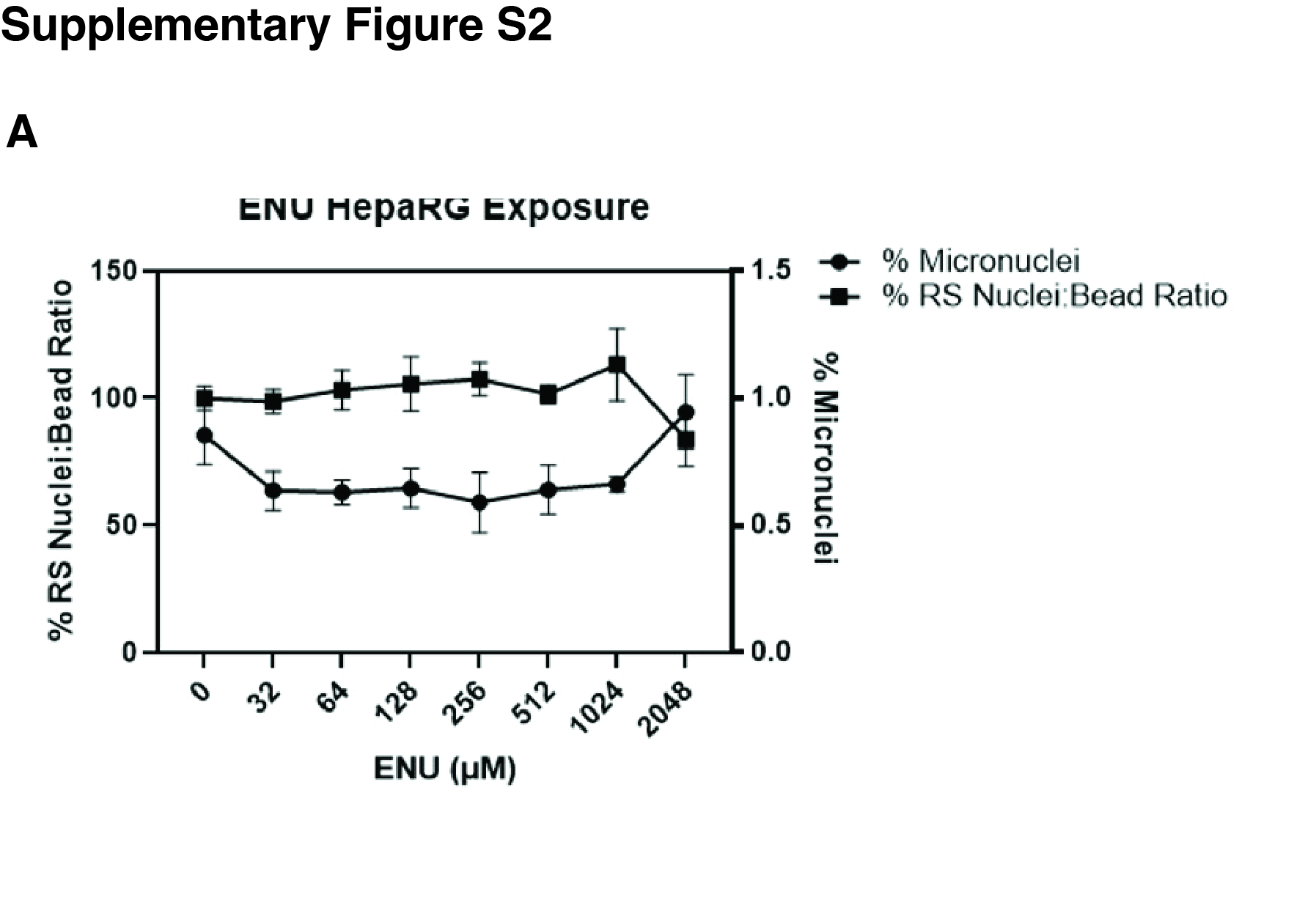

Supplement: Supplementary file 4 [file Image2.tif]

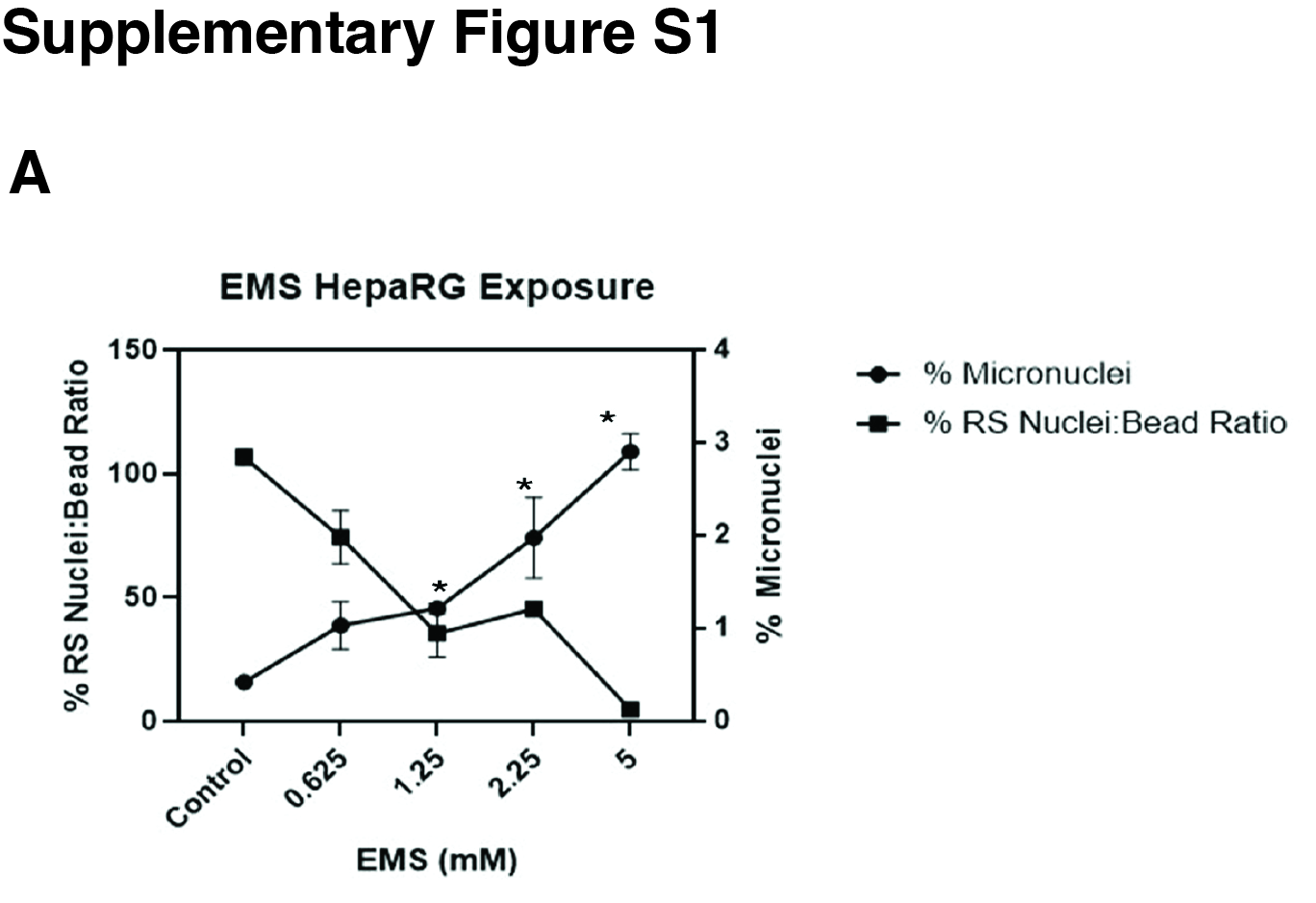

Supplement: Supplementary file 5 [file Image1.tif]

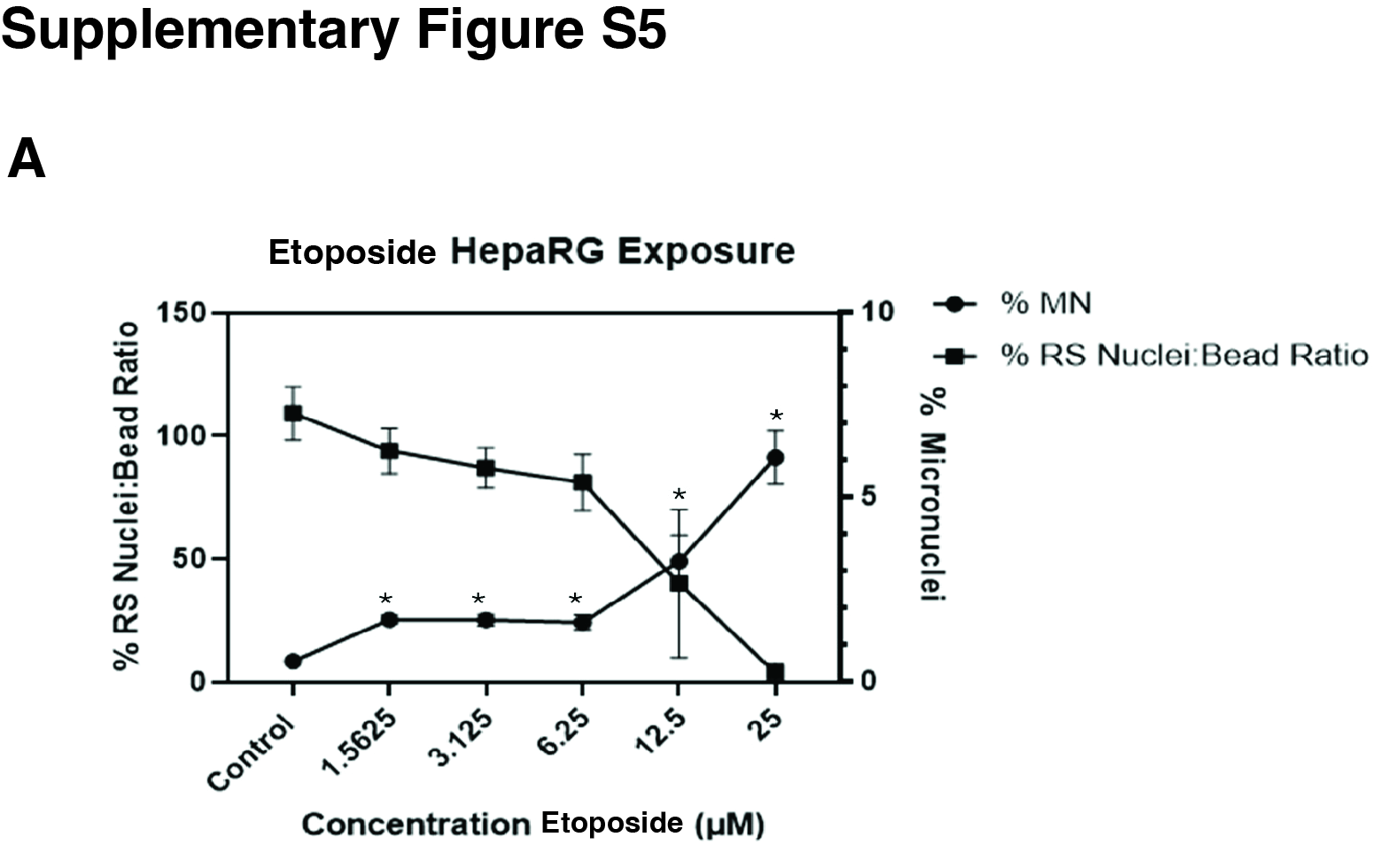

Supplement: Supplementary file 7 [file Image5.tif]
