# Supplementary material for: Error-corrected next-generation sequencing mutagenicity assays in human HepaRG cells as human-relevant genetic toxicology new approach methodology
Source: Front Toxicol. 2025 Sep 15;7:1657189. doi: 10.3389/ftox.2025.1657189 (PMC12477553; doi:10.3389/ftox.2025.1657189)
Supplement: Supplementary file 6 [file DataSheet1.pdf]

SUPPLEMENTARY FIGURE S1
Micronucleus (MN) formation and survival data following EMS exposure. (A) Dose-dependent induction of micronuclei and decrease in relative survival (% RS) across increasing EMS doses in HepaRG™ cells, assessed by flow cytometry. These results were derived from dose-finding experiments and are provided for cytogenetic context.

SUPPLEMENTARY FIGURE S2
Cytogenetic and survival outcomes for ENU-exposed HepaRG™ cells. (A) MN frequency (%MN) and relative survival at increasing ENU concentrations, supporting EC-NGS analysis of point mutation induction.

SUPPLEMENTARY FIGURE S3
BAP-induced cytotoxicity and MN formation in HepaRG™ cells. (A) Cytogenetic responses to BAP exposure, including dose-responsive MN induction and viability changes across doses.

SUPPLEMENTARY FIGURE S4
Cytogenetic analysis of CPA in HepaRG™cells. (A) MN formation and survival outcomes at increasing CPA doses, indicating clastogenic effects not reflected in point mutation frequency.

SUPPLEMENTARY FIGURE S5
Micronucleus data and COSMIC signature profiling for etoposide. (A) %MN and relative survival in cells treated with etoposide, showing strong cytogenetic responses. COSMIC signature results provided in main figure.

SUPPLEMENTARY FIGURE S6
Cytogenetic effects of cisplatin in HepaRG™ cells. (A) MN formation and viability data at multiple cisplatin doses, indicating genotoxic stress. COSMIC signature data shown in main figure.
